# *O-Antigen* decorations in *Salmonella enterica* play a key role in eliciting functional immune responses against heterologous serovars in animal models

**DOI:** 10.3389/fcimb.2024.1347813

**Published:** 2024-02-29

**Authors:** Gianmarco Gasperini, Luisa Massai, Daniele De Simone, Maria Michelina Raso, Elena Palmieri, Renzo Alfini, Omar Rossi, Neil Ravenscroft, Michelle M. Kuttel, Francesca Micoli

**Affiliations:** ^1^GSK, Siena, Italy; ^2^GSK Vaccines Institute for Global Health (GVGH), Siena, Italy; ^3^Department of Chemistry, University of Cape Town, Rondebosch, South Africa; ^4^Department of Computer Science, University of Cape Town, Rondebosch, South Africa

**Keywords:** O-Antigen, GMMA, cross-reactivity, *Salmonella enterica*, conformation, molecular modeling

## Abstract

**Introduction:**

Different serovars of Salmonella enterica cause systemic diseases in humans including enteric fever, caused by S. Typhi and S. Paratyphi A, and invasive nontyphoidal salmonellosis (iNTS), caused mainly by S. Typhimurium and S. Enteritidis. No vaccines are yet available against paratyphoid fever and iNTS but different strategies, based on the immunodominant O-Antigen component of the lipopolysaccharide, are currently being tested. The O-Antigens of S. enterica serovars share structural features including the backbone comprising mannose, rhamnose and galactose as well as further modifications such as O-acetylation and glucosylation. The importance of these O-Antigen decorations for the induced immunogenicity and cross-reactivity has been poorly characterized.

**Methods:**

These immunological aspects were investigated in this study using Generalized Modules for Membrane Antigens (GMMA) as delivery systems for the different O-Antigen variants. This platform allowed the rapid generation and in vivo testing of defined and controlled polysaccharide structures through genetic manipulation of the O-Antigen biosynthetic genes.

**Results:**

Results from mice and rabbit immunization experiments highlighted the important role played by secondary O-Antigen decorations in the induced immunogenicity. Moreover, molecular modeling of O-Antigen conformations corroborated the likelihood of cross-protection between S. enterica serovars.

**Discussion:**

Such results, if confirmed in humans, could have a great impact on the design of a simplified vaccine composition able to maximize functional immune responses against clinically relevant Salmonella enterica serovars.

## 1 Introduction

Different serovars of *Salmonella enterica* cause systemic diseases in humans. In particular, typhoid and paratyphoid fever caused by *S*. Typhi and *S*. Paratyphi A have a high incidence worldwide and coexist in many geographical areas, especially in South-East Asia (Typhoid and Paratyphoid, 2019). Additionally, non-typhoidal *Salmonella* (NTS), together with *S*. Typhi, are a major cause of invasive bacterial disease in sub-Saharan Africa. *S*. Enteritidis and *S*. Typhimurium are responsible for the majority of invasive NTS (iNTS) infections (Collaborators GBDN-TSID, 2019). Infants and children are at particular risk; antibiotics are widely used, but increasing levels of multidrug-resistance limit their effectiveness (Tack et al., 2020). There is broad international consensus on the urgent need for vaccines against systemic *Salmonella* infections. While different vaccines are now available against *S*. Typhi, including Typhoid Conjugate Vaccines (TCV), no vaccines are currently available against paratyphoid fever nor against iNTS and various strategies based on the immuno-dominant O-antigen (OAg) portion of the lipopolysaccharide (LPS) are currently being investigated at the preclinical and clinical level (MacLennan et al., 2014; Tennant et al., 2016).

**Figure 1:**
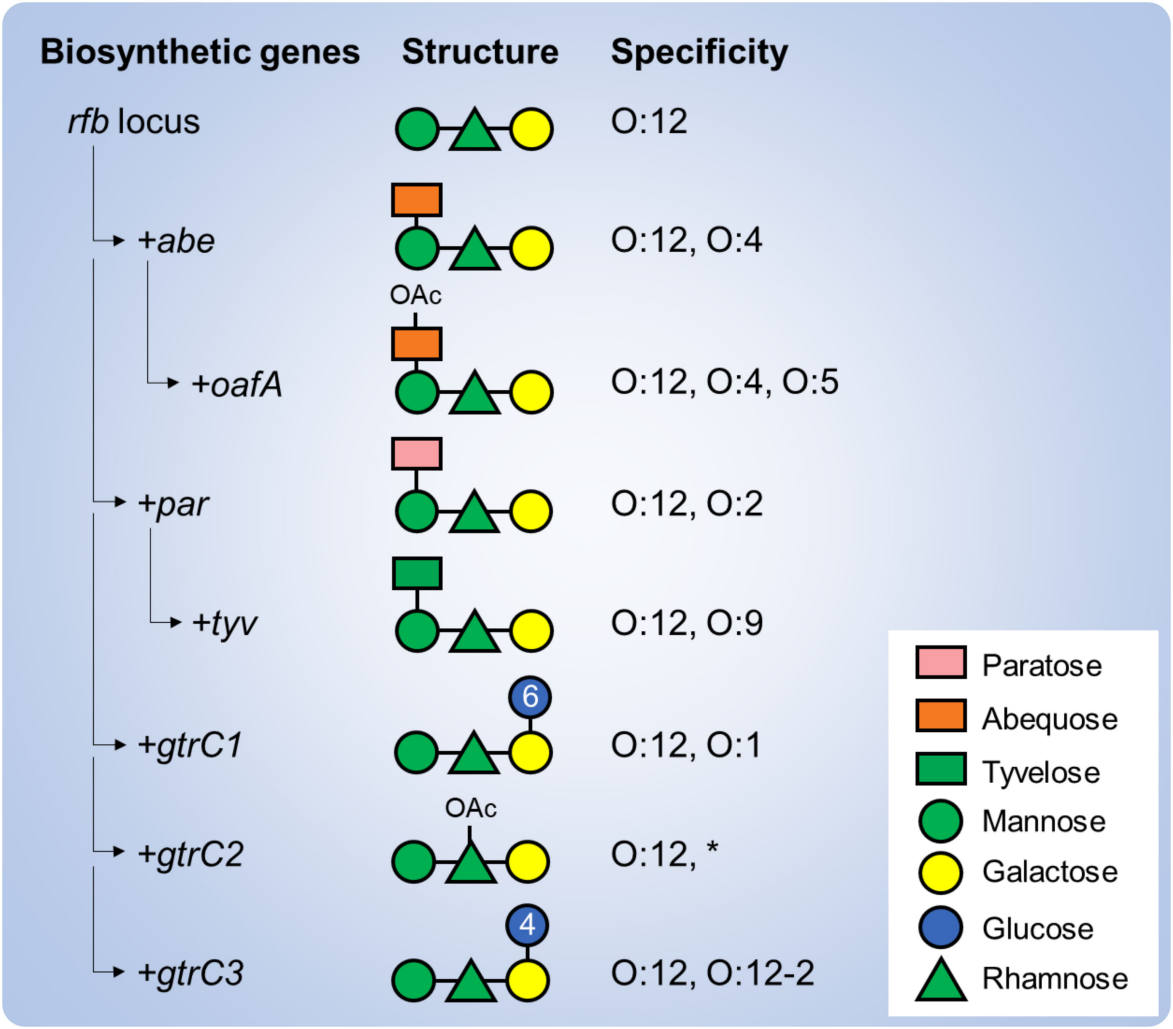
Genetic determinants of the structural and immunological variability of *S. enterica* serovars. Various genes are involved in the biosynthesis of the OAg backbone (*rfb* locus) and addition of the serovar-specific dideoxyhexose residue (*abe*, *par* and *tyv*). Additional genes residing outside the rfb locus, including O-acetyltrasferase (*oafA*, *gtrC2*) and glucosyltransferase (*gtrC1*, *gtrC3*) operons further modify the OAg backbone and confer additional specificities. Numbers in the glucose residues refer to the type of linkage to the galactose residue. *Of note, rhamnose O-acetylation has not been correlated yet to an immune specificity.

The OAg of *S. enterica* serovars Paratyphi A, Typhimurium, Enteritidis and Typhi share a common backbone with a repeating unit (RU) of four sugars: mannose, rhamnose, galactose and a dideoxyhexose linked to mannose. The dideoxyhexose is paratose in *S*. Paratyphi A (conferring the O:2 specificity), abequose in *S*. Typhimurium (conferring the O:4 specificity), and tyvelose in *S*. Enteritidis and *S*. Typhi (conferring the O:9 specificity) (Reeves et al., 2013). Further OAg structural and antigenic modifications have been identified that are driven by genes residing outside of the *rfb* loci (responsible for the OAg biosynthesis) such as glycosyltransferase operons that modify the serotype through glucosylation and O-acetyltransferase genes that modify the serotype through O-acetylation (**Figure 1**) (Micoli et al., 2014; Lanzilao et al., 2015; Ravenscroft et al., 2015; Davies et al., 2013). Among these, 1➔6 glucosylation of the galactose residue has been described in *S*. Paratyphi A as well as in *S*. Typhimurium strains (conferring the O:1 specificity); 1➔4 glucosylation has been described in *S*. Typhiumurium and *S*. Enteritidis strains (conferring the O:12-2 specificity). Moreover, 2-O-acetylation of the abequose residue has been described in *S*. Typhimurium strains (conferring the O:5 specificity), while 2/3-O-acetylation of the rhamnose residue has been described in all three serovars. When shared, such additional specificities could drive cross-reactions, at least to some extent, but knowledge on OAg-mediated cross-reactivity among *S. enterica* serovars remains elusive.

In this study, we aimed to verify the impact of OAg decorations on the induction of functional and cross-reactive immune responses against different *Salmonella* serovars. The Generalized Modules for Membrane Antigens (GMMA) technology was used to investigate the immunogenicity of different OAg structures with the desired features (Gasperini et al., 2021; Gasperini et al., 2022; Raso et al., 2020; De Benedetto et al., 2017). GMMA are outer membrane vesicles naturally released from genetically engineered Gram-negative bacteria. They display the OAg in its natural outer membrane context and have been proposed as a strategy for the development of multi-component vaccines against *Shigella* and iNTS that are currently under evaluation in phase I-II clinical trials (Micoli et al., 2020; Mancini et al., 2021b; Micoli et al., 2022b; Rossi et al., 2023). Importantly, this approach not only allows for rapid generation of *Salmonella* mutants with differing OAg characteristics and simple production of antigens to be tested *in vivo*, but also enables us to obtain a potent immune response for which functionality can be investigated.

Starting from different *Salmonella* strains, we generated *tolR* knock-out mutants of *S*. Paratyphi A, Typhimurium and Enteritidis to increase vesicles formation. GMMA obtained from these strains were used to immunize mice and the resulting antisera were tested for Serum Bactericidal Activity (SBA) against a panel of isolates with different OAg characteristics. Notably, *S*. Paratyphi A GMMA were able to induce significant cross-reactions against heterologous serovars. To further dissect the contribution of the different decorations of *S*. Paratyphi A OAg on the observed cross-reactivity, a panel of isogenic knock-out mutants was generated by removing the enzymes responsible for the OAg O-acetylation or glucosylation. Moreover, an OAg-negative mutant was generated to be included as negative control. The resulting GMMA were then used to immunize mice and rabbits. To assist in rationalizing the observed cross-reactivity, we performed Molecular Dynamics simulations of the OAg displayed on *Salmonella* strains and GMMA, to compare their conformations and exposed epitopes. Taken together, the results from this study contribute to unravelling the role that OAg decorations may have in the induction of cross-protective immune responses against different *Salmonella* serovars.

## 2 Materials and methods

### 2.1 Bacterial strains and generation of mutants

*S*. Paratyphi A strain ED199 (also named NVGH308) is an invasive isolate from Nepal provided by Prof. Stephen Baker (Oxford University Clinical Research Unit, OUCRU, Vietnam) (Dolecek et al., 2008; Dobinson et al., 2017). *S*. Typhimurium strains 2192 and 1418 are animal isolates belonging to the *Salmonella* Genetic Stock Centre (SGSG, University of Calgary, Canada). *S*. Enteritidis strain 618 is an animal isolate obtained by the European Antimicrobial Susceptibility Surveillance in Animals (EASSA), coordinated by the European Animal Health Study Centre, Brussels (CEESA) (de Jong et al., 2013). The clinical *S*. Typhimurium D23580 strain is a representative invasive Malawian isolate belonging to ST313 sequence type isolated from a bacteraemic child, obtained from the Malawi-Liverpool-Wellcome Trust Clinical Research Programme (Blantyre, Malawi) (Kingsley et al., 2009; Okoro et al., 2012). *S*. Typhimurium SL1344 is commonly used laboratory strain isolated by B. Stocker (Hoiseth and Stocker, 1981). *S*. Enteritidis strain CMCC4314 (or ATCC4931) was obtained from the Novartis Master Culture Collection.

*S*. Paratyphi A strain ED199, *S*. Typhimurium strain 2192 and *S*. Enteritidis strain 618 were selected as GMMA-producer strains and genetically engineered accordingly. The null mutations were obtained by replacing the genes of interest with an antibiotic resistance cassette by homologous recombination using the lambda red recombineering system (pSIM18 vector). These mutations did not affect the growth of the strains. The list of all bacterial strains generate, plasmids and primers used is reported in **Supplementary**-**Table S1**. *S*. Paratyphi A strain ED199, *S*. Typhimurium strains 1418, D23580 and SL1344, and *S*. Enteritidis strain CMCC4314 were used as target strains for SBA.

### 2.2 GMMA production and characterization

*Salmonella* GMMA were produced and purified as previously described (Gasperini et al., 2021). Briefly, *Salmonella* GMMA-producing strains were grown in HTMC medium (15 g/L glycerol, 30 g/L yeast extract, 0.5 g/L MgSO_4_, 5 g/L KH_2_PO_4_, 20 g/L K_2_HPO_4_) at 30°C to early stationary phase; bacterial cells were removed through centrifugation and 0.22 µm-filtered supernatants were ultracentrifuged for 2 h at 175,000 *x g*. The GMMA pellet was resuspended in PBS. GMMA total protein content was estimated by micro bicinchoninic acid assay (µBCA) using BSA as a reference. The total OAg amount and sugar composition were determined by high-performance anion-exchange chromatography with pulsed amperometric detection (HPAEC–PAD), after performing acid hydrolysis directly on GMMA. In particular, the OAg amount was quantified based on the detection of rhamnose, as previously described (Micoli et al., 2022a). OAg structures were confirmed by ^1^H-NMR analysis measured with a Bruker AvanceIII 400 spectrometer at 400 MHz and 323 K.

### 2.3 SBA target strains OAg characterization

SBA target strains of *Salmonella* were grown in SBA-like conditions (LB medium, 37°C, 180 rpm shaking) and OAg was purified as previously described (Micoli et al., 2014). Briefly, a solution of 1% (v/v) of AcOH was used to directly resuspend bacterial pellets and acid hydrolysis was performed at 100°C for 2 h. Cells were then centrifuged and the OAg-containing supernatants were 0.22 µm-filtered and desalted using a PD10 column. OAg compositions and structures were finally determined through HPAEC–PAD and ^1^H-NMR.

### 2.4 Animal studies

All animal studies were performed at GSK Animal Facility under the animal project 526/2020-PR 26/05/2020 approved by the Italian Ministry of Health. The studies were ethically reviewed and carried out in accordance with European Directive 2010/63/EEC, the GSK policy on the Care, Welfare and Treatment of Animals, and local animal welfare legislation under Italian authorization. Upon arrival, mice were acclimated in polycarbonate cages type IV (Tecniplast, 2065 cm²) for a period of 5 days. All animals had free access to food (diet ref. A04-10 maintenance from SAFE) and tap water (filtered with a 0.22 µm filter). Nesting material was provided within the cages with nonstructural enrichment material (Nesting cup). Cardboard tunnel and pellets are used as enrichments. Bedding was made of Aspen litter and bedding change was performed once a week or once every two weeks. Upon arrival, rabbits were acclimated in cages (dimension 5112cm² for two rabbits <5kg maximum with a platform size 67x30 cm) with a Noryl perforated tray for a period of 7 days. All animals had free access to food (diet SAFE 112), tap water (filtered with a 0.22 µm filter) and forage bar. Wood stucks are also available in the cages. The perforated tray is changed every day and the cage once a week. Air supplied in housing room was 100% fresh air filtered by EPA filter and the ventilation was at least 20 cycles per hour. The animal room conditions were set as follows: temperature: 20°C (+/- 2°C); humidity: 55% (range from 45-65%) and light/dark cycle: 12h/12 h. The pressure, temperature and relative humidity were recorded continuously by probes. GMMA obtained from *S*. Paratyphi A, Typhimurium and Enteritidis and *S*. Paratyphi A mutants were tested in a single immunogenicity study in mice. GMMA obtained from *S*. Paratyphi A and its mutants were also tested in rabbits. Female, five-week-old CD1 mice (8 per group) were immunized intraperitoneally (IP) with GMMA normalized at 1 µg OAg/dose at day 0 and 28. Female New Zeland White rabbits (6 per group) were immunized intramuscularly (IM) with GMMA normalized at 1 µg OAg/dose at day 0 and 28. OAg-negative GMMA from *S*. Paratyphi A were included as negative control at the same protein dose of the corresponding OAg-positive GMMA. Alhydrogel [Croda] at the final concentration of 0.7 mg/ml was used as adsorbant. Single sera were collected at day 42 from mice and rabbits. Around 15% of the total blood volume was withdrawn from the retromandibular plexus (in mice) or by cardiac puncture (in rabbits) after anesthesia and animals were euthanized before recovery.

Heat inactivated sera (56°C for 30 minutes) were assayed for SBA based on a luminescent readout against a panel of *Salmonella* isolates, as previously described (Necchi et al., 2018). Baby rabbit complement (Cederlane) was used as an exogenous source of complement at 20% for SBA against *S*. Paratyphi A and 50% against the other *Salmonella* strains. Results of the assay were expressed as the IC50, the reciprocal serum dilution that resulted in a 50% reduction of luminescence and thus corresponding to 50% growth inhibition of the bacteria present in the assay 3 hours after the reaction start. Curve fitting for IC50 determination and statistical analysis were performed using GraphPad Prism 7 (La Jolla, CA, USA). The Mann–Whitney U-test was used to compare two groups and a Kruskal–Wallis analysis with *post-hoc* Dunn’s test to compare multiple groups.

### 2.5 Molecular modeling

Individual molecular dynamics (MD) simulations were performed for 1 μs in aqueous solution. The trajectories produced by these simulations were then analyzed and compared, as detailed below.

#### 2.5.1 MD simulation protocol

All simulations were run with the NAMD software package (Phillips et al., 2005), employing CUDA extensions to enable calculation of long-range electrostatic potentials and non-bonded forces on graphics processing units (Stone et al., 2007). Initial structures of 6 RU of each antigen were built with our CarbBuilder software version 1.2.44 which employs the psfgen tool to create files for simulation with CHARMM force fields (Kuttel et al., 2016). The saccharides were modeled with the CHARMM36 additive force field for carbohydrates (Guvench et al., 2011). The starting conformations produced by CarbBuilder were run through 10 000 steps of standard NAMD minimization in vacuum and then placed into a cubic water box with the solvate command from the Visual Molecular Dynamics (VMD) software (Humphrey et al., 1996). The cubic water boxes for all the OAgs had side lengths of 90 Å. The TIP3P model was used to simulate water (Jorgensen et al., 1983).

Each system was then gradually heated through a protocol of 5 K incremental temperature reassignments from 10 K to 300 K, with 500 steps of NAMD minimization and 8 000 steps of MD after each temperature reassignment. MD simulations were then run for 1 μs.

In each simulation, equations of motion were integrated using a Leap-Frog Verlet integrator with a step size of 1 fs and periodic boundary conditions. Simulations were performed under isothermal-isobaric (nPT) conditions at 300 K maintained using a Langevin piston barostat (Zhang et al., 1995) and a Nose-Hoover thermostat (Nosé and Klein, 2006). Long-range electrostatic interactions were treated using particle mesh Ewald (PME) summation, with k = 0.20 Å-1 and a 1 Å PME grid spacing. Non-bonded interactions were truncated with a switching function applied between 12.0 and 15.0 Å to groups with integer charge. The 1–4 interactions were not scaled, in accordance with the CHARMM force field recommendations.

For all simulations, structures were collected at intervals of 250 fs for analysis.

#### 2.5.2 MD data analysis

Analysis of the simulations used time series frames 25 ps apart. Molecular conformations extracted from the MD simulations were depicted with VMD.

End-to-end distance, *r*, for each of the antigens were calculated from the C2 atom in the αDMan residues at the reducing and non-reducing ends of the chain.

Conformations from all MD simulation trajectories were clustered using VMD’s internal *measure cluster* command to generate conformational families. Clustering analysis used time series frames 250 ps apart, discarding the first 125 ns as equilibration. For clustering analysis simulation conformations were first aligned on the ring atoms of the residues in repeat units 2, 3, 4 and 5 of the 6 unit chain. Then all conformations were clustered into families according to a root mean square deviation (rmsd) fit (cutoff of 6 Å) to the saccharide ring atoms.

## 3 Results

### 3.1 Analytical characterization of the OAg displayed on GMMA derived from *S*. paratyphi A, typhimyrium and enteritidis

*S*. Paratyphi A, Typhimurium and Enteritidis wild-type strains were genetically manipulated to obtain overblebbing phenotypes through knock-out of the *tolR* gene. GMMA were purified from each *Salmonella* strain and analytically characterized, as reported in **Table 1** and **Supplementary Figures 1, 2**.

**Table 1:** Analytical characterization of the OAg displayed on *S. enterica* GMMA.

GMMA	OAg/protein ratio [HPAEC-PAD/μBCA]	% OAg Glc [HPAEC-PAD]	Glc linkage (Ref)	% OAg OAc [^1^H-NMR]	OAc linkage [^1^H-NMR]
**ParA GMMA** (from strain ED199)	0.40	63	1➔6 Gal (Ravenscroft et al., 2015)	48	Rhamnose
**STm GMMA** (from strain 2192)	1.01	22	1➔4 Gal + 1➔6 Gal (Micoli et al., 2014)	86	Abequose
**SEn GMMA** (from strain 618)	1.14	nd	–	9	Rhamnose

nd, not detected.

*S*. Paratyphi A GMMA (ParA GMMA) showed a lower OAg/protein ratio compared to *S*. Typhimurium and *S*. Enteritidis GMMA (STm GMMA and SEn GMMA). In contrast, ParA GMMA showed the highest OAg glucosylation level (linked 1➔6 to the galactose residue) and the highest rhamnose O-acetylation level.

### 3.2 Immunological investigation of cross-reactivity among *Salmonella* serovars

With the aim to investigate the functionality of immune responses elicited after GMMA vaccination, different *S. enterica* strains were selected to be used as a representative SBA panel maximizing the diversity of the displayed OAg structures. These strains were grown in SBA-like conditions and the displayed OAg were fully characterized, as reported in **Table 2** and **Supplementary Figures 3, 4**.

**Table 2:** Analytical characterization of the OAg displayed on *S. enterica* SBA target strains.

Strain	% OAg Glc [HPAEC-PAD]	Glc linkage (Ref)	% OAg OAc [^1^H-NMR]	OAc linkage [^1^H-NMR]
**ParA ED199**	98	1➔6 Gal (Ravenscroft et al., 2015)	67	Rhamnose
**STm 1418**	64	1➔4 Gal + 1➔6 Gal (Micoli et al., 2014)	69	Abequose
**STm D23580**	nd	–	69 + 81	Abequose + Rhamnose
**STm SL1344**	nd	–	75	Abequose
**SEn CMCC4314**	nd	–	nd	nd

nd, not detected.

In particular, three different STm strains were selected due to their specific OAg features. Compared to STm SL1344, which shows OAg O-acetylation on abequose only and no OAg glucosylation, STm 1418 has a similar OAg O-acetylation pattern but shows a high level of OAg glucosylation (which can occur in either the 4 or 6 position on the galactose residue), while STm D23580 carries an additional OAg O-acetylation on the rhamnose residue. ParA strain ED199 is the parental strain to the GMMA-producing strain, therefore the OAg displayed has comparable structural modifications to that displayed on ParA GMMA. The SEn CMCC4314 strain has no measurable OAg glucosylation or O-acetylation.

ParA, STm and SEn GMMA were normalized at 1 µg OAg dose, adsorbed on Alhydrogel and used to immunize mice intraperitoneally twice at 4-week intervals. Sera collected at day 42 were then analyzed for functionality by SBA against the described panel of strains.

**Figure 2:**
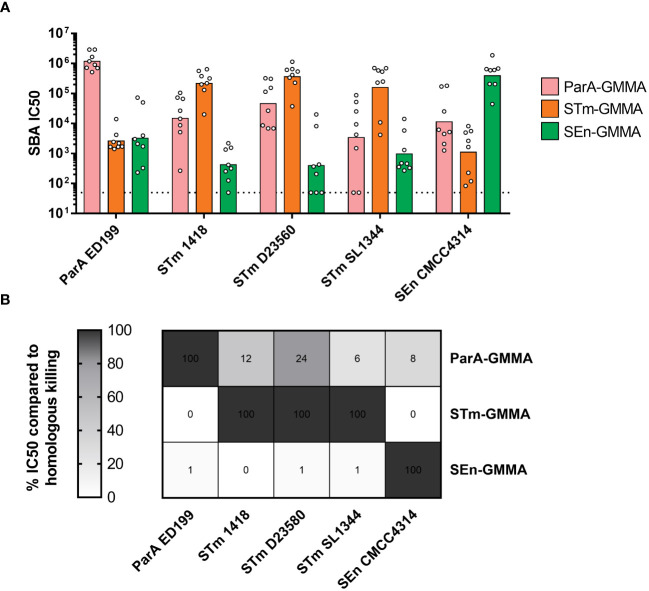
Bactericidal activity of sera raised in mice by *S. enterica* GMMA. **(A)** Summary graphs of SBA titers raised by ParA GMMA (pink), STm GMMA (orange) SEn GMMA (green) against different *S. enterica* serovars (x asis) with individual IC50 levels (dots) and geometric mean (bars). The dotted line corresponds to non-bactericidal titers (pre-immune sera). **(B)** Evaluation of cross-reactivity in terms of relative percentace of killing of heterologous serovars compared to homologous killing.

As expected, all GMMA were able to elicit high levels of bactericidal antibodies against the homologous serovars (**Figure 2A**). Interestingly, a certain level of bactericidal activity was measured with all sera against all strains (**Figure 2A**). To interpret the data, homologous killing was set as the maximum level (100%) of bactericidal activity observed in this experimental settings, and heterologous killing was calculated accordingly (**Figure 2B**). ParA GMMA was the only one able to elicit a heterologous bactericidal activity (higher than 10%) against STm strains 1418 and D23580, as well as a lower though measurable activity against STm SL1344 and SEn CMCC4314 (**Figure 2B**). In contrast, STm GMMA and SEn GMMA appeared to elicit much stronger bactericidal antibodies against the homologous compared to the heterologous serovars.

### 3.3 Analytical and immunological characterization of GMMA derived from *S*. Paratyphi A and its isogenic mutants

To further explore the observed cross-reactivity, the *S*. Paratyphi Δ*tolR* strain was further mutated to remove the *gtrC1* gene (responsible for OAg glucosylation), the *gtrC2* gene (responsible for OAg O-acetylation) or the *rfbP-U* genes (responsible for the biosynthesis of the OAg chains). **Table 3** and **Supplementary Figures 1, 2** report the characterization of the resulting GMMA.

**Table 3:** Analytical characterization of the OAg displayed on *S.* Paratyphi A GMMA and its isogenic mutants.

GMMA	OAg/protein ratio [HPAEC-PAD/μBCA]	% OAg Glc [HPAEC-PAD]	Glc linkage (Ref)	% OAg OAc [^1^H-NMR]	OAc linkage [^1^H-NMR]
**ParA GMMA**	0.40	63	1➔6 Gal (Ravenscroft et al., 2015)	48	Rhamnose
**ParA GMMA ΔGlc**	0.37	nd	–	55	Rhamnose
**ParA GMMA ΔOAc**	0.36	69	1➔6 Gal (Ravenscroft et al., 2015)	nd	–
**ParA GMMA ΔOAg**	–	nd	–	nd	–

nd, not detected.

The analytical characterization confirmed the two expected OAg phenotypes, with complete abolishment of either OAg glucosylation, OAg O-acetylation or OAg biosynthesis in general. Interestingly, the mutation added did not have any impact on the remaining OAg features, nor on the GMMA OAg/protein ratio.

Once again, GMMA were normalized at 1 µg OAg dose, adsorbed on Alhydrogel and used to immunize mice IP or rabbits IM, twice at 4-week intervals. Sera collected at day 42 were analyzed for functionality by SBA against the aforementioned panel of *Salmonella* isolates.

**Figure 3:**
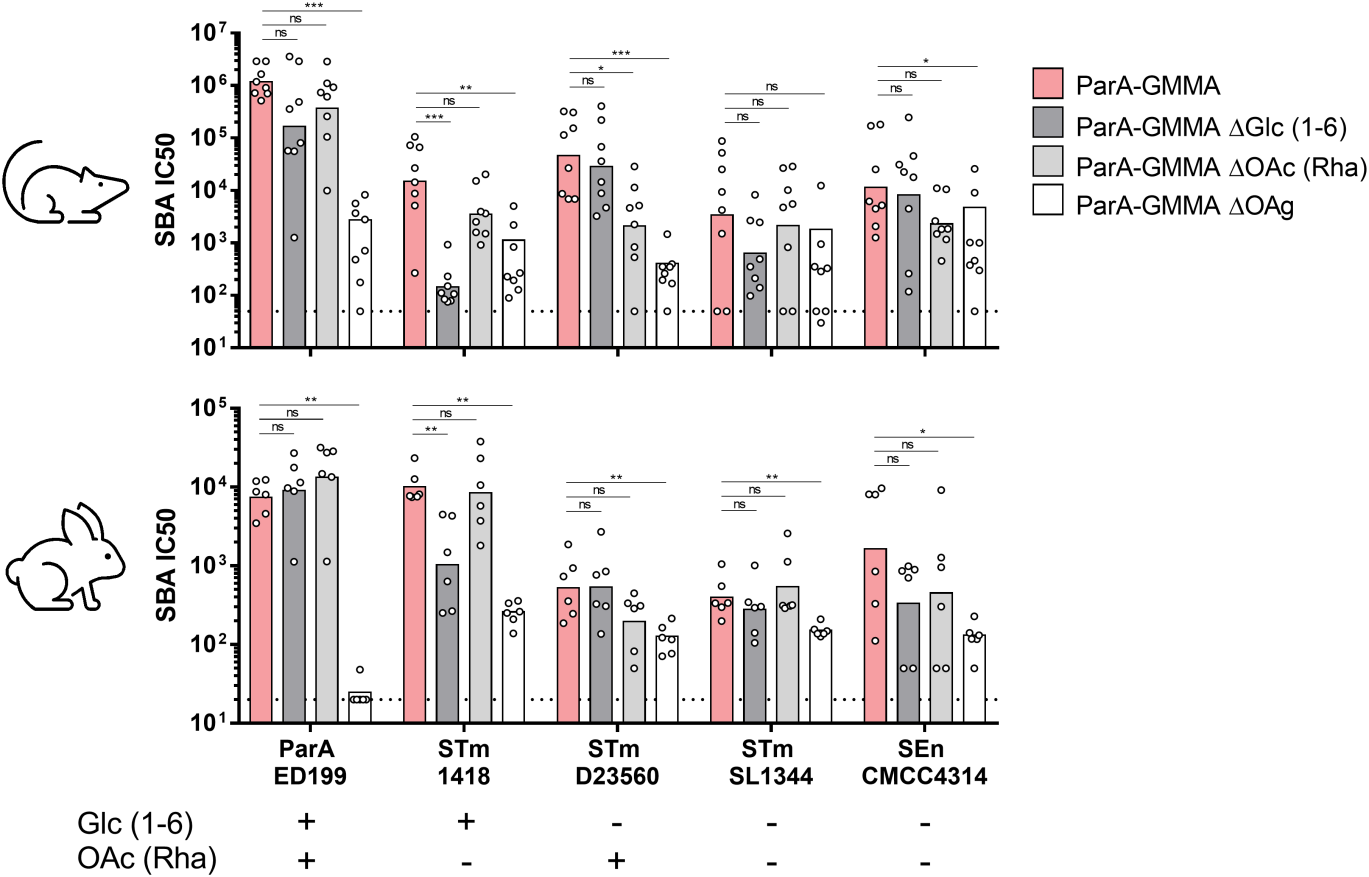
Bactericidal activity of sera raised in mice and rabbits by S. Paratyphi A GMMA. Summary graphs of SBA titers raised by different ParA GMMA against different *S. enterica* serovars reporting individual IC50 levels (dots) and geometric mean (bars) in mice or rabbits. The dotted line corresponds to non-bactericidal titers (pre-immune sera). The Mann–Whitney U-test was used to compare ParA GMMA and ParA GMMA ΔOAg groups, while the Kruskal–Wallis analysis with post-hoc Dunn’s test to compare ParA GMMA, ParA GMMA ΔGlc and ParA GMMA ΔOAc groups (*: p<0.03; **: p<0.002; ***: p<0.0002).

The impact of OAg glucosylation and O-acetylation was investigated in terms of SBA titers elicited against homologous and heterologous strains (**Figure 3**). Interestingly, both ParA GMMA ΔGlc and ΔOAc elicited similar bactericidal activity against *S*. Paratyphi A strain ED199 compared to ParA GMMA in mice and rabbits. When tested against *S*. Typhimurium strain 1418, sera raised against ParA GMMA ΔGlc, but not against ParA GMMA ΔOAc, were significantly less bactericidal than sera raised against ParA GMMA both in mice and rabbits. When tested against *S*. Typhimurium strain D23580, sera raised against ParA GMMA ΔOAc, but not against ParA GMMA ΔGlc, were significantly less bactericidal than sera raised against ParA GMMA in mice but not in rabbits. Finally, when tested against *S*. Typhimurium strain SL1344 and S. Enteritidis strain CMCC4314, no differences were observed between ParA GMMA ΔGlc, ParA GMMA ΔOAc and ParA GMMA both in mice and rabbits. The bactericidal activity of sera raised against ParA GMMA ΔOAg was significantly lower than that measured in sera raised against ParA GMMA for all strains used, with the exception of STm SL1344 tested with mice sera.

### 3.4 Molecular modeling

Individual MD simulations were performed for the OAg previously described and characterized either on GMMA or on the SBA target strains. The modeled antigens are reported in **Table 4** and represent the most abundant molecular species (i.e. O-acetylation and glucosylation levels lower than 40% were not modeled).

**Table 4:** *S. enterica* O-antigens modeled in this work.

OAg source (GMMA/strain)	Most abundant OAg molecular structure (common backbone residues in bold)
ParA GMMA/ParA ED199	**→2)**[αDPar(1→3)]**αDMan(1→4)αLRha**3OAc**(1→3)**[αDGlc(1->6)]**αDGal**(→
STm GMMA/STm SL1344	**→2)**[αDAbe2Ac(1→3)]**αDMan(1→4)αLRha(1→3)αDGal**(→
SEn GMMA/SEn CMCC4314	**→2)**[αDTyv(1→3)]**αDMan(1→4)αLRha(1→3)αDGal**(→
STm 1418	**→2)**[αDAbe2Ac(1→3)]**αDMan(1→4)αLRha(1→3)**[**α**DGlc (1->6)]**αDGal**(→
STm D23580	**→2)**[αDAbe2Ac(1→3)]**αDMan(1→4)αLRha**3OAc**(1→3)αDGal**(→

Of note, αLRha3OAc was modeled even if both αLRha3OAc and αLRha2OAc are observed in ^1^H-NMR spectra.

**Figure 4:**
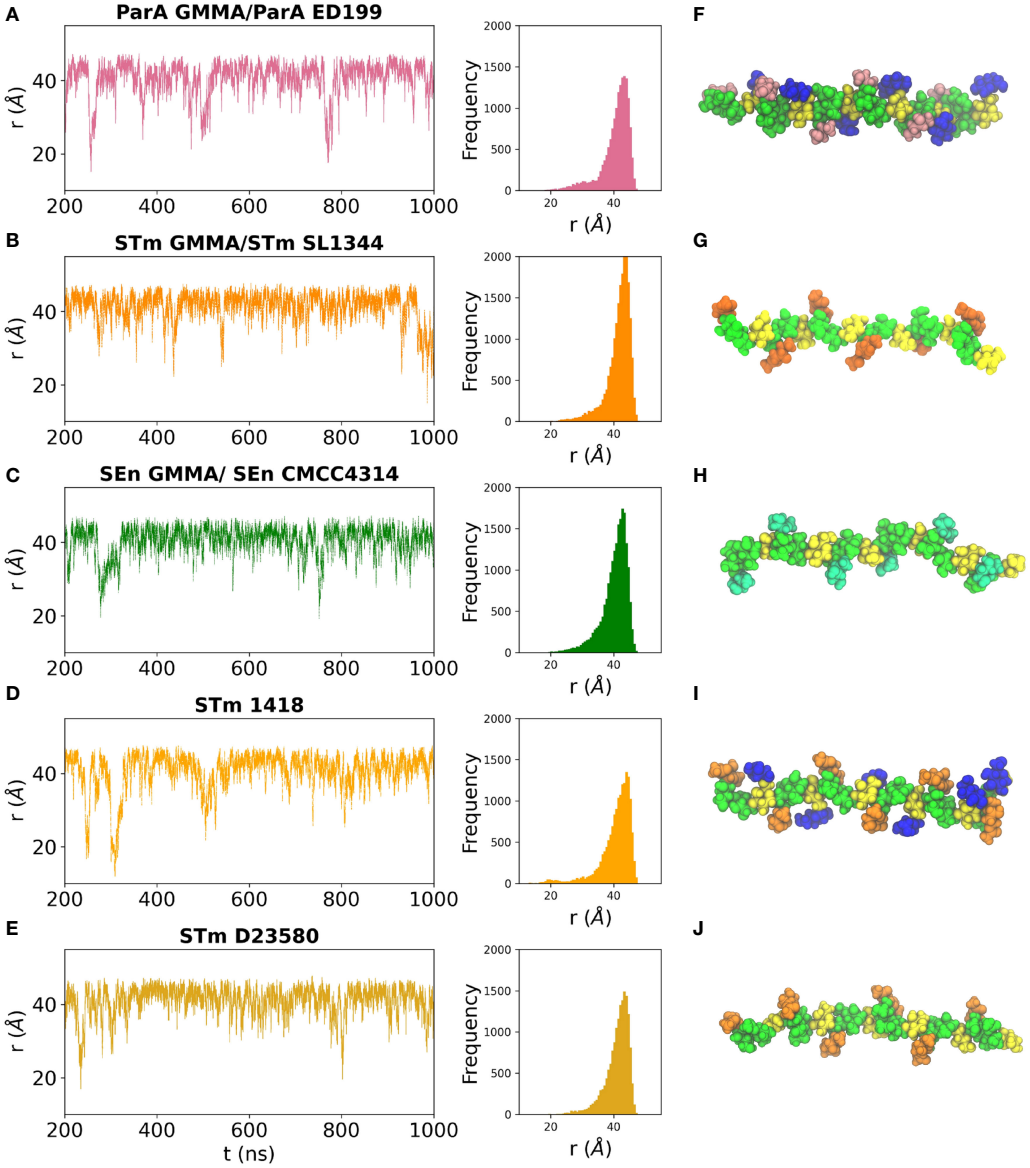
Time series plots and corresponding histograms for the simulation trajectories (left panels) and snapshots of the dominant conformation for each OAg (right panels) are shown for the OAg displayed on: **(A, F)** ParA GMMA / ParA ED199; **(B, G)** STm GMMA / STm SL1344; **(C, H)** SEn GMMA / SEn CMCC4314; **(D, I)** STm 1418 and **(E, J)** STm D23580. OAg residues colored as follows: aDMan green, aLRha/aLRha3OAc green, aDGal yellow, aDGlc blue, aDAbe2Ac orange, aDTyv cyan, αDPar pink.

**Figure 5:**
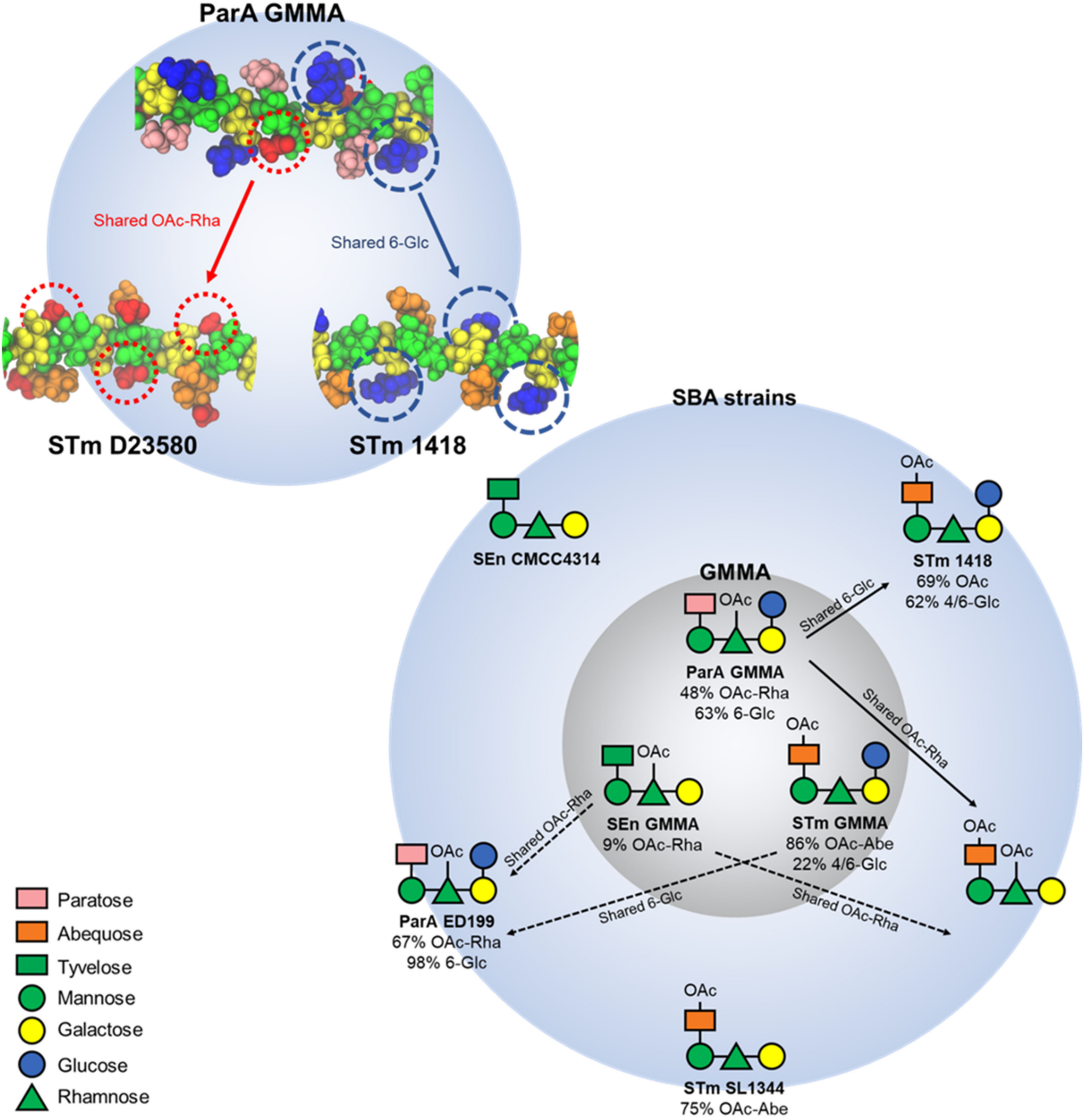
Structure-function correlation based on immunological data and molecular modelling. The structure of the OAg variants displayed on GMMA and on the different SBA target strains. Solid arrows show structurally-predicted and *in vivo* validated cross-reactivities. The shared epitopes that rationalise this cross-reactivity are highlighted in the structure on top right. Dotted arrows show structurally-predicted cross-reactivities that were not validated by functional data, most likely due to very low levels of O-acetylation or glucosylation on GMMA-displayed OAg.

The dynamic behavior of a linear carbohydrate can be measured by the time series of the end-to-end distance, *r*, of the chain, throughout a simulation (**Figure 4**). Graphs of *r* for the simulations of 6 RU of each of the five antigens and the corresponding histograms (**Figures 4A–E**) indicate similar chain extensions and dynamics for all the molecules. All of the antigens are primarily in an extended conformation (**Figures 4F–J**): glucosylation and O-acetylation do not significantly increase the conformational differences between the strains. Sharp spikes in the time series indicate that the occasional bends that do occur in the chains are of short duration. The side chain substitutions that differentiate the strains are very exposed on the backbone and thus potentially available for antigen binding. In ParA OAg (**Figure 4F**), the αDPar and αDGlc side chains are in close proximity on the same side of the antigen backbone. In STm OAg (**Figure 4G**), the O-acetyl substitution on αDAbe2Ac is highly exposed on the end of the residue; the αDTyv residue in SEn OAg (**Figure 4H**), although similarly exposed, is clearly smaller in size. The addition of a 4-linked αDGlc in STm (**Figure 4I**) makes a large difference to the antigen surface as compared to the addition of a 3-linked OAc (**Figure 4J**). This is illustrated in **Figure 5**: the OAg displayed on ParA GMMA and STm D23580 share a common epitope of an exposed O-acetyl substitution on the backbone rhamnose residue. Similarly, the OAg displayed on ParA GMMA and STm 1418 share the exposed 6-Glc epitope.

## 4 Discussion

The work of Kauffmann and White first established a typing scheme for *Salmonella* based on serology of the O and H antigens. This scheme has since been expanded and refined, and now includes 46 OAg and 114 H antigens in 2557 serovars, each with a unique combination of O antigen and H antigens (Grimont and F-XJWccfr, 2007). *Salmonella* is divided into 2 species, *S. enterica* and *S. bongori*, and *S. enterica* is divided into six subspecies, with *S. enterica subsp. enterica* causing approximately 99% of *Salmonella* infections in humans and warm-blooded animals.

The variation in OAg structures is used to define *S. enterica* serogroups and within *S. enterica subsp. enterica*, the most common O-antigen serogroups are A, B, C1, C2, D and E. In particular, serogroup A is characterized by the O:2 specificity conferred by a paratose side branch and includes serovar Paratyphi A; serogroup B is characterized by the O:4 specificity conferred by an abequose side branch and includes serovar Typhimurium and serogroup D is characterized by the O:9 specificity conferred by a tyvelose side branch and includes serovars Typhi and Enteritidis (Reeves et al., 2013).

Aside from the serological classification, OAg variation also determines the specificity of immunity to *Salmonella*, induced by natural infection or vaccination. Indeed, surface-accessible OAg are major targets of protective anti-*Salmonella* antibodies and passive protection studies demonstrated that IgG or IgM directed against the immunodominant and serovar-specific O:2, O:4 or O:9 O-epitopes plays an important role in disease prevention (Carlin et al., 1987). However, less is known about the functional role of other non-specific O-epitopes, which arise from further modifications of the common OAg backbone, e.g. O-acetyl groups or differently linked glucose moieties. Such modifications can be shared between serovars and therefore contribute to cross-reactivity.

In this study, we aimed to investigate the ability of *S. enterica* OAg to elicit functional immune responses against homologous and heterologous serovars, employing the GMMA platform not only as a delivery system but also as a rapid tool to dissect the contribution of individual OAg modifications such as glucosylation and O-acetylation. In particular, *S*. Paratyphi A strain ED199, *S*. Typhimurium strain 2192 and *S*. Enteritidis strain 618 were selected as GMMA-producer strains as these isolates are used as OAg source for different vaccines currently in clinical trials, including GMMA-based and glycoconjugate candidates (NCT05480800, NCT05613205, ISRCTN51750695).

STm GMMA and ParA GMMA were characterized by the well described O:5 and O:1 O-epitopes respectively, due to the O-acetyltransferase gene *oafA* and a P22-like glucosyltransferase operon *gtrC* family I. ParA GMMA were also characterized by high levels of rhamnose-O-acetylation, due to a BTP1-like O-acetyltransferase gene *gtrC* family II. Low levels of glucosylation were measured for STm GMMA, while low levels of O-acetylation were measured for SEn GMMA, suggesting the presence of different OAg-modifying enzymes controlled by genetic phase-variation (Broadbent et al., 2010; Kintz et al., 2017).

Following mice immunization, the resulting antisera were tested for their bactericidal activity against a panel of homologous and heterologous *Salmonella* strains. No cross-reactivity was observed between STm and SEn GMMA, indicating that the common OAg backbone is either not largely immunogenic or not accessible to antibody binding, as previously described. On the contrary, ParA GMMA elicited sera with high bactericidal activity, not only against the homologous *S*. Paratyphi A strain ED199 but also against heterologous strains, particularly *S*. Typhimurium strains D23580 and 1418.

Characterization of such strains also confirmed the presence of high rhamnose-O-acetylation levels in STm D23580, and high glucosylation levels in STm 1418, suggesting that the observed cross-reactivity could be linked to shared OAg modification. To better investigate this, ParA GMMA were further mutated in order to abolish glucosylation on galactose or O-acetylation on rhamnose. The immune responses elicited by this set of mutated GMMA were compared to that elicited by the original ParA GMMA, both in mice and rabbits.

Interestingly, while ParA OAg glucosylation or O-acetylation appeared to have no major role in the serum bactericidal activity against the homologous *S.* Paratyphi A strain ED199, they were confirmed as major determinants of cross-reactivity towards *S.* Typhimurium strains 1418 and D23580, respectively. This confirmed the original hypothesis and clearly correlated the observed cross-reactivity to a shared epitope composed of glucosylated-galactose (also known as O:1) or a shared epitope composed of O-acetylated-rhamnose.

Finally, to rule out that the observed reactivity was determined by other antigens displayed on GMMA, an additional construct was obtained to abolish the OAg biosynthesis. Interestingly, OAg-negative GMMA proved able to elicit a bactericidal immune responses against different *Salmonella* serovars, indicating that antibodies not targeting the OAg (including anti core and anti-proteins antibodies) are produced upon immunization and are able to determine complement-mediated killing, at least to some extent. This is in contrast to what was previously observed with *Shigella* GMMA (Mancini et al., 2021a; Mancini et al., 2023), indicating that in the case of *Salmonella*, protein antigens play a role in the immune response elicited by GMMA. Moreover, when comparing sera elicited by ParA GMMA and ParA GMMA ΔOAg in their bactericidal activity against *S*. Enteritidis strain CMCC4314, a statistically significant difference was observed both in mice and rabbits, implying that cross-reactivity between ParA and SEn is indeed mediated by the OAg but not by shared O-acetylation or glucosylation. Specific conformations of the OAg backbone or low-affinitiy antibodies recognizing the dideoxyhexose residue might be involved.

Molecular modeling revealed that the OAg have very similar extended conformations, with the side chains exposed for possible antibody binding. The cross-protection between ParA GMMA and STmD23580 can be explained as they share a common epitope of an exposed O-acetyl substitution on the backbone rhamnose residue, while ParA GMMA and STm 1418 share the highly exposed 6-Glc epitope. In general, this suggests that the OAg sidechain substitutions are the probable main cause of cross-protection (or the lack thereof) between the *S. enterica* strains, through the exposure of common epitopes for antibody binding.

In conclusion, this work sheds light on the immunological cross-reactivities between *S. enterica* OAg structures which are not mediated by the immunodominant and serogroup-specific OAg epitopes. Multi-valent strategies are currently being explored to overcome the serovar-specific nature of the immune response elicited against *Salmonella* OAg. For example, a bivalent approach targeting *S*. Enteritidis and *S*. Typhimurium (ISRCTN51750695), and a trivalent approach targeting *S*. Enteritidis, *S*. Typhimurium, and *S*. Typhi (NCT05480800) have been described and are undergoing clinical evaluation. The findings presented here and summarized in **Figure 5** will be of critical importance to maximise the impact of OAg-based *Salmonella* vaccines and to estimate the breadth of vaccine coverage.

## Data availability statement

The original contributions presented in the study are included in the article/**Supplementary Material**. Further inquiries can be directed to the corresponding author.

## Ethics statement

All animal studies were performed at GSK Animal Facility under the animal project 526/2020-PR 26/05/2020 approved by the Italian Ministry of Health. The studies were ethically reviewed and carried out in accordance with European Directive 2010/63/EEC, the GSK policy on the Care, Welfare and Treatment of Animals, and local animal welfare legislation under Italian authorization. The study was conducted in accordance with the local legislation and institutional requirements.

## Author contributions

GG: Conceptualization, Methodology, Supervision, Writing – original draft, Writing – review & editing, Data curation. LM: Data curation, Methodology, Writing – review & editing. DD: Data curation, Methodology, Writing – review & editing. MR: Data curation, Methodology, Writing – review & editing. EP: Data curation, Methodology, Writing – review & editing. RA: Data curation, Methodology, Writing – review & editing. OR: Data curation, Methodology, Supervision, Writing – review & editing. NR: Supervision, Writing – review & editing. MK: Conceptualization, Methodology, Writing – original draft, Writing – review & editing, Data curation. FM: Conceptualization, Supervision, Writing – review & editing.
