# Supplementary material for: O-Antigen decorations in Salmonella enterica play a key role in eliciting functional immune responses against heterologous serovars in animal models
Source: Front Cell Infect Microbiol. 2024 Feb 29;14:1347813. doi: 10.3389/fcimb.2024.1347813 (PMC10937413; doi:10.3389/fcimb.2024.1347813)
Supplement: Supplementary file 1 [file DataSheet_1.docx]

Supplementary Material

**Supplementary Table 1.** List of all strains and primers used in the present study.

| **Strain name/genotype** | | | **Notes** | |
| --- | --- | --- | --- | --- |
| *S. enterica* serovar Paratyphi A ED199 | | | Used for SBA | |
| *S. enterica* serovar Paratyphi A ED199 *tolR::cat* | | | Used to produced ParA GMMA | |
| *S. enterica* serovar Paratyphi A ED199 *tolR::cat gtrC1::aph* | | | Used to produced ParA GMMA ΔGlc | |
| *S. enterica* serovar Paratyphi A ED199 *tolR::cat gtrC2::aph* | | | Used to produced ParA GMMA ΔOAc | |
| *S. enterica* serovar Paratyphi A ED199 *tolR::cat rfbU-P::aph* | | | Used to produced ParA GMMA ΔOAg | |
| *S. enterica* serovar Typhimurium 2192 | | | **-** | |
| *S. enterica* serovar Typhimurium 2192 *tolR::aph* | | | Used to produced STm GMMA | |
| *S. enterica* serovar Typhimurium 1418 | | | Used for SBA | |
| *S. enterica* serovar Typhimurium SL1344 | | | Used for SBA | |
| *S. enterica* serovar Typhimurium D23580 | | | Used for SBA | |
| *S. enterica* serovar Enteritidis 618 | | | **-** | |
| *S. enterica* serovar Enteritidis 618 *tolR::aph* | | | Used to produced SEn GMMA | |
| *S. enterica* serovar Enteritidis CMCC4314 | | | Used for SBA | |
| **Primer name** | **5’-3’ Sequence** | | | **DNA template** |
| *tolR* KO_F | accgccaggcgtttaccgttagcgagagcaacaaggggtaagccatggcc**GTGTAGGCTGGAGCTGCTTC** | | | pKD4 (aph);  pKD3 (cat) |
| *tolR* KO_R | acccgctctctttcaagcaagggaaacgcagatgtttagataggctgcgt**CATATGAATATCCTCCTTAG** | | |  |
| *gtrC1* KO_F | GGCGATTATATATACATTTCCTTTATTGACACATCAATCATTTTTCGTTG**GTGTAGGCTGGAGCTGCTTC** | | | pKD4 (aph) |
| *gtrC1* KO_R | TACTGAATCCCGTTCGTTTAAAGCTGTTGCTAATGGGAAAATTTACATTG**CATATGAATATCCTCCTTAG** | | |  |
| *gtrC2* KO_F | GGTTTTATCTGTGGTAATATTCCATTATTTCCCATCATTATTGCCGGGTG**GTGTAGGCTGGAGCTGCTTC** | | | pKD4 (aph) |
| *gtrC2* KO_R | AATCAGGACCATTACCAACTCTTGTAAGACATCCACTTTCGTTACATAAT**CATATGAATATCCTCCTTAG** | | |  |
| *rfbP-U* KO_F | CAGATTTTACGCAGGCTAATTTATACAATTATTATTCAGTACTTCTCGGTA**GTGTAGGCTGGAGCTGCTTC** | | | pKD4 (aph) |
| *rfbP-U* KO_R | TCGTCCTTGTGATAATCGCTATACAAGCATGAATGGAAAAACGGAGAGCT**CATATGAATATCCTCCTTAG** | | |  |
| **Vector name** | | **Ref** | | |
| pSIM18 | | [Chan W. et al., Nucleic Acids Research, Volume 35, Issue 8, 15 April 2007, Page e64] | | |

**Supplementary Figure S1:** ^1^H NMR spectra of OAg extracted from ParA GMMA (A), ParA GMMA ΔOAc (B), ParA GMMA ΔGlc (C), STm GMMA (D) and SEn GMMA (E).

**
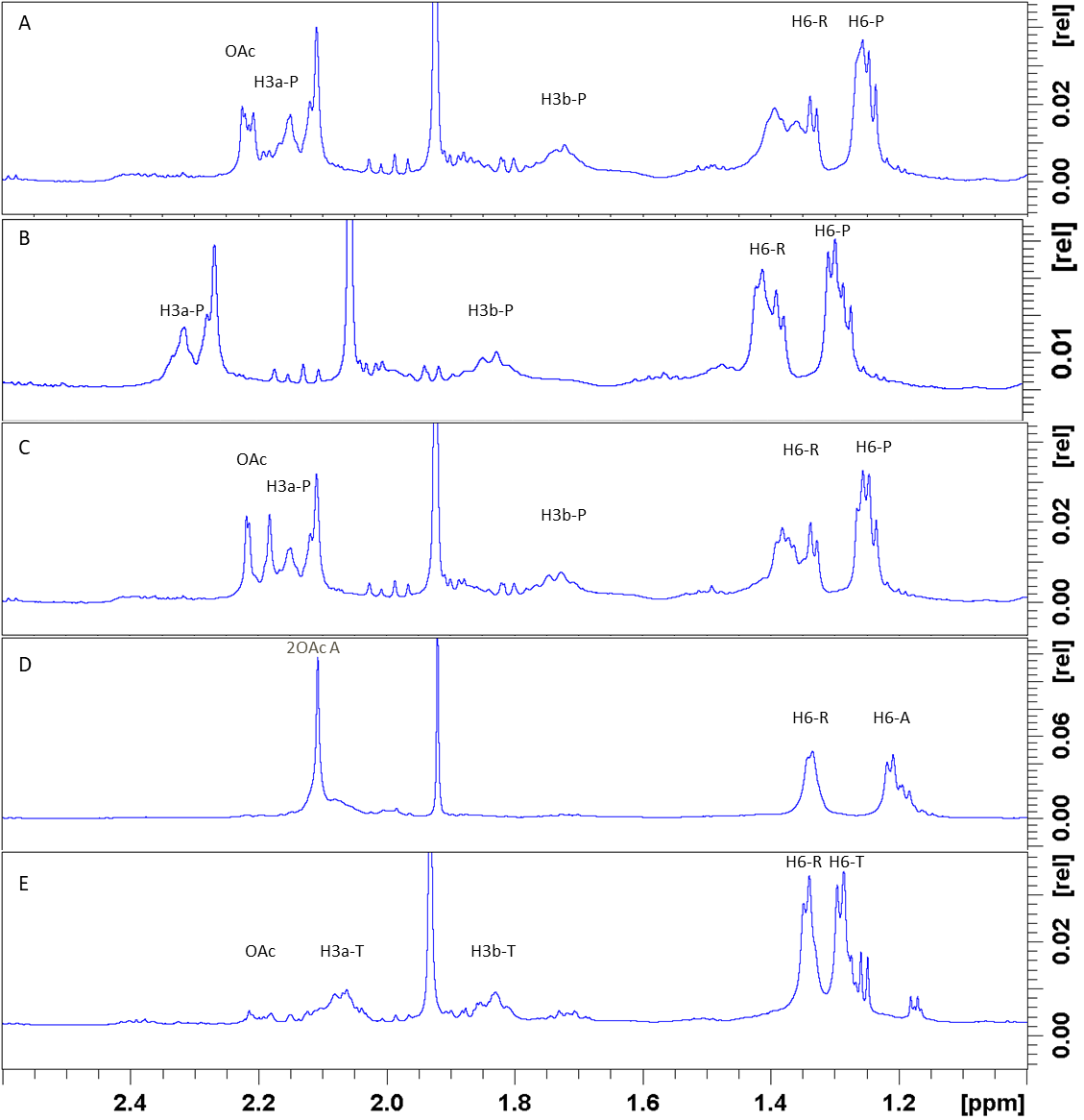
Supplementary Figure S2:** ^1^H NMR spectra with zoom on the O-Acetyl region of OAg extracted from ParA GMMA (A), ParA GMMA ΔOAc (B), ParA GMMA ΔGlc (C), STm GMMA (D) and SEn GMMA (E). Some assignments are labeled based on reference [Ravenscroft, N., et al. 2015; Micoli, F., et al. 2014; Snyder, D. S., et al. 2006. The sugar residues are labeled upper case (P= Par, A = Abe, T= Tyv, R = Rha)

**Supplementary Figure S3:** ^1^H NMR spectra of OAg extracted from *S.* Paratyphi A ED199 (A), *S.* Typhimurium 1418 (B), D23580 (C) SL1344 (D) and *S.* Enteritidis CMCC4314 (E) wild type strains.

**
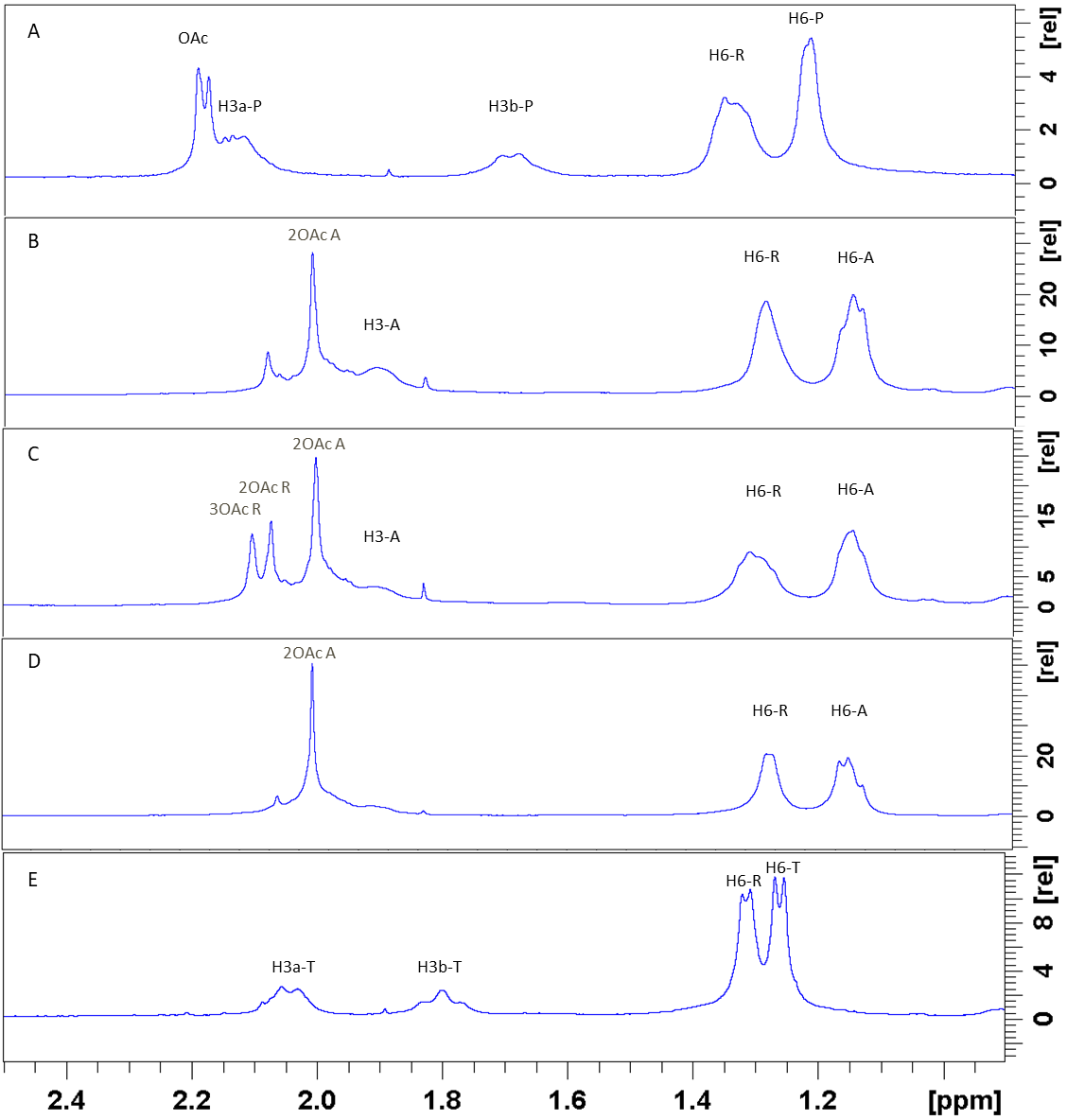
Supplementary Figure S4:** ^1^H NMR spectra with zoom on the O-Acetyl region of OAg extracted from *S.* Paratyphi A ED199 (A), *S.* Typhimurium 1418 (B), D23580 (C) SL1344 (D) and *S.* Enteritidis CMCC4314 (E) wild type strains. Some assignments are labeled based on reference [Ravenscroft, N., et al. 2015; Micoli, F., et al. 2014; Snyder, D. S., et al. 2006. The sugar residues are labeled in upper case (P= Par, A = Abe, T= Tyv, R = Rha)
